# Adaptable polyaryletherketones (PAEKs) with competing crosslinking and crystallisation mechanisms

**DOI:** 10.1038/s41598-024-51231-3

**Published:** 2024-01-05

**Authors:** Nan Yi, Adam Chaplin, John Grasmeder, Oana Ghita

**Affiliations:** 1 Faculty of Environment, Science and Economy, University of Exeter, Exeter, EX4 4QF UK; 2 Victrex Manufacturing Limited, Hillhouse International, Thornton Cleveleys, Lancashire, FY5 4QD UK

**Keywords:** Engineering, Materials science

## Abstract

Driven by the need to make high temperature thermoplastic polymers more processable and expand the range of applications, this study reports on the properties of a novel PAEK material developed by Victrex (Thornton Cleveleys, UK) which is capable of undergoing crosslinking or crystallisation, two competing processes that can be adapted via specific processing temperature and time conditions. The uniqueness of this PAEK material resides in its manufacturing approach, where the crosslinkers are incorporated during the polymerisation process, and its distinct properties, including a controllable viscosity that can be tuned from low to high to allow its application in complex manufacturing processes, such as thermoplastic carbon fibre manufacturing.

## Introduction

High strength-to-weight ratio and high service temperature make polyaryletherketones (PAEKs) sought-after materials in highly demanding applications^1^. They are extensively used in the aerospace and automotive industries, particularly in the composite form reinforced with carbon fibre^2^. Despite the versatile processing techniques that can be used for PAEKs, such as injection moulding, extrusion, and compression moulding, the high processing temperatures and high melt viscosities pose continuous challenges for manufacturers, especially when producing carbon fibre reinforced PAEK (CF/PAEK) components^3^.

To transform from the initial polymer state into the final thermoplastic composite products, polymers undergo a two-step process involving the preparation of prepregs and subsequent fabrication. Both steps require low viscosity for optimal results. Composite prepreg tapes are manufactured by impregnating carbon fibres with thermoplastic polymer. To ensure effective impregnation, resins are commonly adhered to the fibre surface using solvent-based or water-based powder application methods^4^. These approaches rely on low viscosity polymer solutions to sufficiently wet the fibre surface and facilitate effective impregnation. However, the presence of residual solvents poses challenges as they have negative impact on the overall mechanical properties and are often difficult to remove^4,5^. To address these issues, alternative methods such as film stacking^6^ and dry powder impregnation^7^ have been developed. In those new methods, low melt viscosity remains desirable for impregnating fibres. When fabricating the final parts, viscosity also plays a critical role in forming the prepregs into the desired shape. Excessively low viscosity can result in resin migration^8^, while excessively high viscosity can hinder consolidation and restrict fibre movement^5^. Therefore, achieving an appropriate viscosity level is crucial for successful thermoplastic composite part manufacturing.

In the first step, full and uniform coverage of the carbon fibre by the thermoplastic is essential to achieve uniform mechanical performance and minimise defects including voids. A traditional way to ensure good wet out is to improve flow by increasing the processing temperature. Another traditional method to promote the impregnation is to dissolve PAEKs in high-boiling solvents such as molten diphenylsulfone (DPS) to temporarily reduce the viscosity^9^. Both methods are hazardous with a risk of thermal degradation and fire. Moreover, they are expensive due to the cost of high temperature equipment or solvent, and the requirement of high-level processing control and monitoring, making it impractical for large scale commercial manufacturing.

To tackle this challenge, Manolakis et. al.^10^ blended polyetheretherketone (PEEK) with low molecular weight (MW) thioether ketone macrocyclic oligomers. These oligomers are stable at the beginning of the impregnation process, acting as plasticisers to reduce the viscosity of molten PEEK, then the macrocyclic oligomers go through spontaneous ring-opening polymerisation triggered when the processing temperature is raised above 350 °C. It is reported that the initial melt viscosity of PEEK blend with 20% macrocyclic oligomers could be lowered by about an order of magnitude (when tested at 350 °C and 100 s^-1^), and reverted to the original PEEK viscosity after ring-opening polymerisation. In theory, the polymerised high-MW poly(thioether-ketone) should not affect the overall mechanical strength of the PEEK blend. However, no follow-up experiments have been done to provide mechanical data.

Deliberately inducing crosslinking in a PEEK matrix has been investigated since the beginning of PEEK being developed. In 1982, Sutter et. al.^11^ first synthesised crosslinked PEEK by inserting biphenylene units onto the aromatic backbone as reaction sites. The researchers observed a remarkable transformation in crosslinked PEEK, where it changed its solubility behaviour from being soluble in concentrated sulfuric acid to becoming completely insoluble. Crosslinking at temperatures between the glass transition temperature (Tg) and the melt temperature (Tm) has been achieved by compounding PEEK with sulphur^12^, resulting in an increased Tg and melt viscosity. In 1988, Thompson and Farris^13^ successfully crosslinked PEEK via imine formation at carbonyl groups. While this method doubled the Tg and greatly improved the mechanical stability of the material at high temperatures, it made the material impossible to process or characterise. Hence in 2010, Yurchenko et. al.^14^ proposed a two-stage procedure to moderately crosslink PEEK by inserting aromatic imines as rigid crosslinkers, aiming to produce PEEK elastomer for high temperature applications. Other methods include thermal annealing^15^ and electron and ion irradiation^15^. However, none of the abovementioned methods is suitable for fabricating CF/PAEK components^10^.

**Figure 1:**
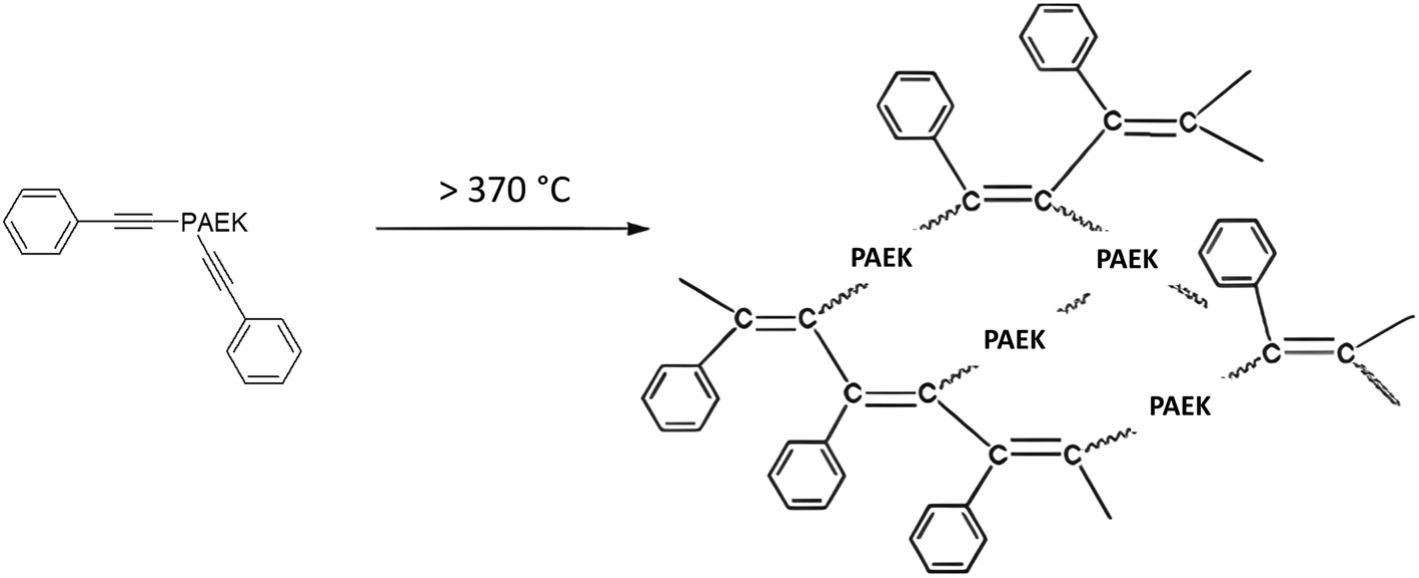
Crosslinking reaction mechanisms adapted from^16^.

This study introduces a novel class of PAEK-B2 materials, incorporating phenylethynyl end-capped crosslinkers. The molecular structure of the crosslinker involves an aromatic ring bonded to the phenyl structure. Phenylethynyl acts as a crosslinkable moiety, enabling the formation of crosslinks between polymer chains through thermal processes, see Fig. 1^16^. The triple bonds react with each other on heating without generating gaseous by-products^17^. This results in the development of a three-dimensional network structure^18^. When compared to other crosslinking agents, using phenylethynyl for crosslinking has benefits including lower reaction temperatures and shorter reaction times^16^. Additionally, incorporating phenylethynyl at the end-cap position allows the molecular chain to extend, making it possible to achieve a high molecular weight at the final product and thus obtaining high mechanical properties.

Incorporating these crosslinkers manufactured by NEXAM offers several advantages over existing commercially available PAEK materials^17^. Firstly, it retains substantial crystallinity at the initial form, making it possible to tune the final crystallinity. Furthermore, the polymerisation process of incorporating the crosslinker does not generate any undesirable by-products such as salts or gases, eliminating the need for complex downstream processing. Most importantly, the crosslinking agents facilitate melt processing of the PAEK material at lower temperatures and within a wide processing window, allowing for enhanced control and efficiency in manufacturing.

In this study, the properties of the new reactive Victrex developmental PAEK with controllable melt viscosity and crystallinity were reported, which could be used to facilitate processing CF/PAEK components. This modified Victrex PAEK (coded as PAEK-B2) initially has a low MW and a low melt viscosity at the temperature below 360 °C to ensure good flow but have the ability to crosslink and extend the molecular chains above the trigger temperature of 360 °C. The level of crosslinking could be adjusted by controlling the temperature and the duration of this post heat treatment step. After crosslinking, the final mechanical properties are expected to be comparable to normal PEEK with high MW and high melt viscosity.

## Materials and experimental

### Material and sample preparation

Developmental grade PAEK-B2 powder was supplied by Victrex. The low MW PAEK was loaded with 3 mol % reactive phenylethynyl crosslinkers during the polymerisation stage. One-millimetre-thick sheets were compression moulded at three different pressing temperatures, i.e., 330 °C, 400 °C, and 450 °C. A pressure of 65 MPa was hold over the 100 mm by 100 mm mould area for a pressing time ranging from 10 to 60 min. The level of crosslinking is adjusted by the combination of the pressing temperature and holding time. At 330 °C pressing temperature, a 10 min holding time was selected to allow fabrication of a test sample and avoid crosslinking. At 400 °C a series of holding times were selected: 10, 20, 30, and 60 min. Although the 450 °C temperature is expected to trigger degradation, for the purposes of understanding the two competing processes (i.e., crystallisation and crosslinking) and the full extent of the processing window, 5 min and 20 min holding times were chosen at this temperature.

### Characterisation methods

#### Thermal analysis

Thermal properties of the prepared samples were determined by differential scanning calorimetry (DSC) performed on a Mettler Toledo DSC1. Approximately 5 mg mass of moulded sample was encapsulated in a standard Aluminium pan with an Aluminium lid. Heat and cooling ramps were performed at 10 °C/min under a 50 ml/min Nitrogen flow. Crystallinity was calculated via computing the ratio of enthalpy of melting to the theoretical enthalpy of 100% crystalline PAEK, i.e., 130 J/g^19^.

#### X-ray diffraction measurements

The overall crystallinity was analysed using wide-angle X-ray diffraction (WAXD) measurements. In this study, the intensity spectrum along the Bragg angle was obtained from a Bruker D8 Advance XRD diffractometer with Cu Kα radiation (λ = 0.154 nm). All measurements were performed in the “Locked Couple” mode with a 2θ angle of 9 to 45°. The beam was generated at 40 kV and 40 mA. The diffraction profiles were acquired in a “Step Scan” method at a scan speed of 0.5 s/step.

#### Rheological measurements

Melt viscosities were measured by a TA Instruments DHR-1 equipped with 25 mm parallel plate geometry. Circular disks of 1 mm thick were cut out from the compression moulded sheet. Temperature sweep tests were conducted to identify the trigger temperature of crosslinking reaction. Viscosities were measured from 280 and 400 °C at 5 °C/min with an angular frequency of 1 Hz, purged by a continuous air flow. A 0.5 N constant axial force was used to control the gap distance.

#### Mechanical analysis

Mechanical properties of the prepared samples were evaluated by tensile testing performed on a Shimadzu standard tensile/compression machine with a 20 kN load cell. ISO 527-1BA tensile bars were cut out from the compression moulded sheet. The testing speed was 5 mm/min for tensile strength and elongation at break. In total five repeats were tested for each moulding condition.

### Crosslinking degree evaluation

In this study, the degree of crosslinking was calculated from both DSC and XRD measurements. XRD provides static measurements on the sample as moulded, whereas DSC provides dynamic measurements accounting for further thermal reactions. The employment of both methods aimed to determine their respective effectiveness and suitability for evaluating the degree of crosslinking.

**Figure 2:**
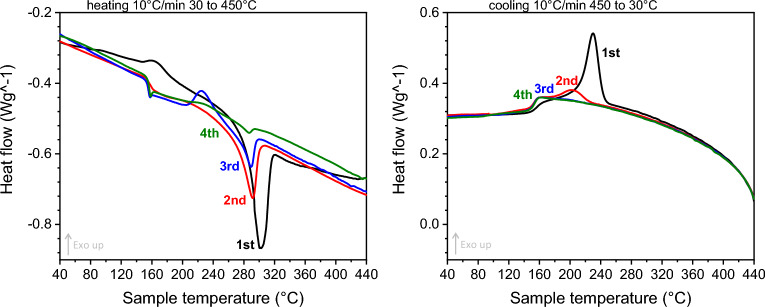
DSC thermograms of PAEK-B2 undergoing four sequential heating and cooling ramps.

Crosslinking reactions of PEEK are known to compete with the crystallisation process, often resulted in suppressed exothermic recrystallisation peaks and endothermic melting peaks^13,14,20^. It indicates the disruption of the molecular chain regularity, which is required to form polymeric crystallites. A DSC scan with four heating and cooling ramps in sequence was performed to reveal further information on the competition of the two processes, see Fig. 2. During the first cycle, PAEK-B2 exhibits strong melting and recrystallisation peaks, marking the initial highly crystalline state. The melting endotherm and the recrystallisation exotherm decrease as the number of cycle increases, revealing the disruption of crystalline microstructure by crosslinking. It is evident that the presence of exothermic recrystallisation peak is less sensitive to the level of crosslinking than the endothermic melting peak, similar to the results reported in^14^. For this reason, the ratio of the melting entropy of the processed sample to the entropy of virgin PAEK-B2 is used in this study to calculate the degree of crosslinking:

1 $${C}_{DSC}\%=1-\frac{\Delta {H}_{f}({T}_{m})}{\Delta {H}_{f,ref}}$$

where $\Delta {H}_{f}({T}_{m})$ is the enthalpy of fusion at the melting point for a specific pressing temperature/time profile, $\Delta {H}_{f,ref}$ refers to the reference enthalpy of melting of the 0% crosslinked PAEK-B2. The reference enthalpy, 34.82 J/g, was determined from the average enthalpy of four repeating tests with heating ramps up to 330 °C. Rheological evidence shows the crosslinking reaction is not triggered below 330 °C as presented in the "Rheological analysis".

In the same manner, the degree of crosslinking can be determined using the XRD profiles. First, it is necessary to calculate the absolute crystallinity using the following equation:

2 $${X}_{C}=\frac{\sum {S}_{C}}{{S}_{A}+\sum {S}_{C}}$$

where ${S}_{A}$ is the area of the amorphous peak, and ${S}_{C}$ is the area of the crystalline peak. Subsequently, the degree of crosslinking can be calculated by comparing it to the absolute crystallinity of 0% crosslinked PAEK-B2, denoted as ${X}_{C,ref}$:

3 $${C}_{XRD}\%=1-\frac{{X}_{C}}{{X}_{C,ref}}$$

### Thermogravimetric analysis

The degradation behaviour of PAEK-B2 was examined by thermogravimetric analysis (TGA) using a Mettler Toledo DSC/TGA 1+. Two protocols were developed for this investigation. The non-isothermal protocol includes a heat ramp of 10 °C/min from 30 to 800 °C. Meanwhile, the isothermal protocol includes a rapid heating ramp of 100 °C/min from 30 °C to the isothermal temperatures, followed by a one-hour isothermal segment. All tests were performed under a 100 ml/min Nitrogen flow.

## Results and discussion

### Timescale for crosslinking

**Figure 3:**
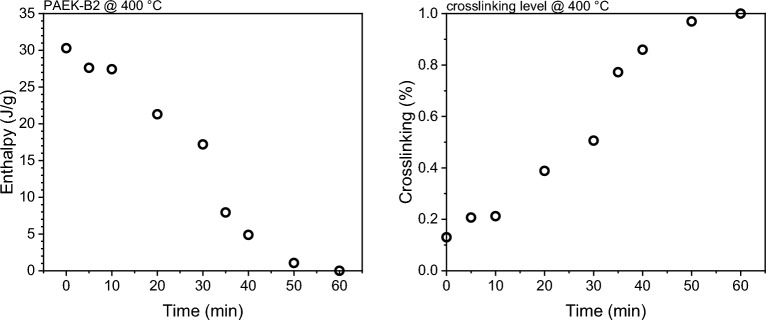
Enthalpy and level of crosslinking as a function of processing time at 400 °C.

Figure 3 presents the degree of crosslinking as a function of processing time at 400 °C. The evaluation of crosslinking was conducted through isothermal tests in DSC at 400 °C, with the isothermal times ranging from 0 to 60 min. In Fig. 3, the initial degree of crosslinking is observed at 0.1, possibly indicating the commencement of crosslinking during the heating ramp to reach 400 °C. The relationship between degree of crosslinking and isothermal time is non-linear, featuring an S-shaped correlation. This pattern indicates a gradual rise of degree of crosslinking at the beginning, followed by a rapid increase around 30 min, ultimately plateauing around 50 min.

The trend in Fig. 3 confirms the benefits this grade has for thermoplastic manufacturing processes (e.g. automated fibre placement and thermoforming), slow crosslinking at the beginning of the process which accelerates with time. The mobility of the molecular chains at the beginning of the process promotes intimate contact and interdiffusion. The processing window (i.e. 50 min) is comparable with thermosets manufacturing and wider than most of the thermoplastic processes^21^.

Another potential application of the crosslinkable PAEK-B2 material is to fabricate functionally graded material (FGM) structures. While previous studies have successfully controlled the local crystallinity of PEEK to achieve FGM in 3D printing^22,23^, preliminary experiments in this study indicate a significant challenge in the context of additive manufacturing processes such as material extrusion (MEX) and powder bed fusion (PBF). In these processes, the residence time above the crosslinking temperature is limited to less than a second^19^, whereas effective crosslinking requires a reaction time ranging from few minutes to one hour, as illustrated in Fig. 3. This temporal constraint poses a considerable obstacle to achieving a controlled gradient of crosslinking level necessary for fabricating FGM structures via additive manufacturing.

### Degree of crosslinking degree and colouration measurements

**Figure 4:**
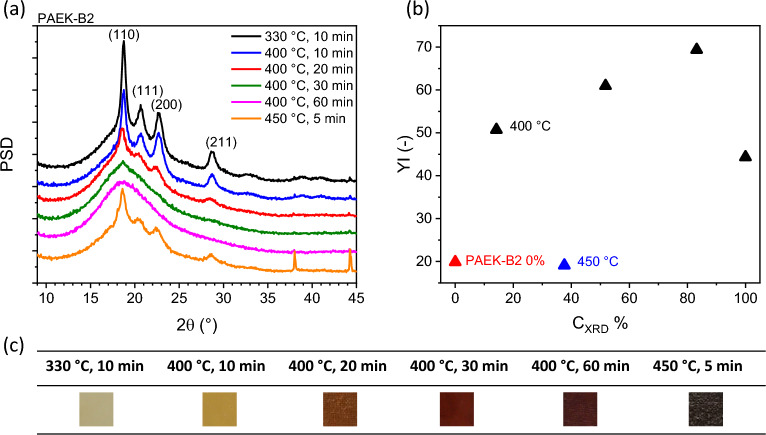
(**a**) Wide-angle X-ray spectra; (**b**) yellowness indexes; and (**c**) colouration for PAEK-B2s.

The degrees of crosslinking obtained via DSC and XRD are summarised in Table 1. The sample compressed at 330 °C for 10 min was used as the reference in Eqs. (1) and (3). The XRD spectra presented in Fig. 4 reaffirm the inverse relationship between the degree of crosslinking and the degree of crystallinity.

**Table 1:** Summary of compression moulding conditions and the degrees of crosslinking.

Compression moulding temperature (°C)	Holding time (min)	${C}_{DSC}\%$	${C}_{XRD}\%$
330	10	0	0
400	10	23.6	14.2
400	20	39.1	51.8
400	30	39.6	83.2
400	60	44.9	100
450	5	27.1	37.6
450	20	56.9	N/A

As observed in Table 1, the degree of crosslinking increases with longer holding times at a temperature of 400 °C. At a temperature of 450 °C, although the crosslinking reaction is initiated, the photo in Fig. 4 shows signs of severe oxidation and degradation, evident from the sample’s colour turning into charcoal at 450 °C. It is noteworthy that the degree of crosslinking obtained from DSC measurements consistently differs from the degrees accessed through XRD measurements. This is not uncommon^24,25^, the disparity may be attributed to the fundamental differences between the two methods employed and possibly due to additional reorganisation processes taking place during the heating ramp prior to melting in DSC measurement.

The yellowness index is a numerical measurement that quantifies the degree of yellowness in a sample. It is commonly used in polymer industry to access the colour stability and degradation of materials. According to the ASTM standard, the yellowness index is typically determined by the CIE tristimulus values from a colorimeter or spectrophotometer^26^. The higher the yellowness index value, the more yellow the sample appears. In 2020, a methodology to convert the red, green, and blue values of the RGB colour to the CIE tristimulus values was proposed^27^. The method is adopted in this study to calculate the yellowness index presented in Fig. 4b, accompanied by photos illustrating the colouration of the samples in Fig. 4c.

Figure 4 illustrates the relationship between the yellowness index and the degree of crosslinking. The figure displays that non-crosslinked PAEK-B2 (compression moulded at 330 °C) and degraded PAEK-B2 (compression moulded at 450 °C) exhibit similarly low yellowness indices. However, at compression mould temperature of 400 °C, the yellowness index progressively increases with the degree of crosslinking, reaching a peak around 83.2% of crosslinking. Interestingly, the yellowness index then decreases for the fully crosslinked PAEK-B2, which may be attributed to degradation. The colour change could be potentially used as a method of inline monitoring of the degree of crosslinking. However, more in-depth studies should be performed to confirm and create robust datasets.

### Degradation behaviours

**Figure 5:**
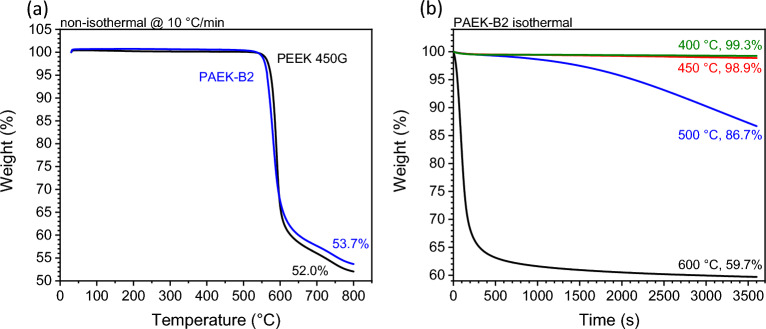
The weight change of PAEK-B2 under non-isothermal and isothermal conditions.

Figure 5 displays the thermal degradation behaviours of PAEK-B2. The onset degradation temperature of PAEK-B2, determined through non-isothermal testing as in Fig. 5a, is compared to PEEK 450G™ as a benchmark. It can be seen that the degradation behaviours of PAEK-B2 closely resembles that of PEEK 450G™, with both materials exhibiting onset degradation temperatures around 550 °C. Furthermore, a comparable weight loss is observed in both materials upon reaching 800 °C. The similarity on degradation behaviours confirms the suitability of PAEK-B2 for conventional composite manufacturing processes, which already employed common PAEKs such as PEEK 450G™.

Figure 5b presents the isothermal TGA results. At 400 °C for one hour, the minimal weight loss percentage of 0.7% suggests the suitability of this processing temperature for composite manufacturing using PAEK-B2. Remarkably, the weight loss only slightly increases to 1.1% at 450 °C after one hour. The modest change implies that the observed colour changes at 450 °C for 5 min in Fig. 4c results from a limited amount of degradation. This observation is further supported by XRD crystalline peak shown in Fig. 4a, where the presence of crystalline peaks indicates that the material is not fully degraded thus no crystals could form. A substantial weight loss is observed at 500 °C (13.3%) and 600 °C (40.3%) over the same duration. The data reveals a non-linear relationship between degradation and processing temperatures, suggesting an acceleration of the degradation reaction at higher temperatures.

### Rheological analysis

**Figure 6:**
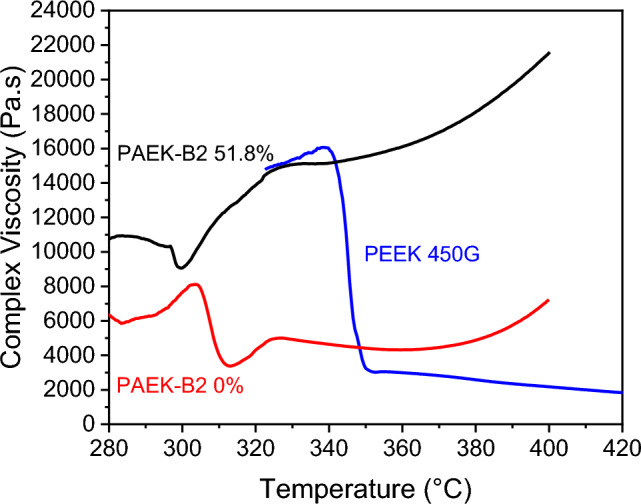
Complex viscosity of Victrex PEEK 450G™, PAEK-B2 0% and 51.8% crosslinked, as a function of temperature at 5 °C/min and 1 Hz.

Figure 6 displays the viscosity of non-crosslinked (red solid line prepared at 330 °C for 10 min) and partially crosslinked PAEK-B2 (black solid line prepared at 400 °C for 20 min) as a function of temperature. The degrees of crosslinking were calculated according to Eq. (3) introduced in "Crosslinking degree evaluation". The viscosity of Victrex PEEK 450G™ was measured and added for comparison. Tests on PAEK-B2 started from 280 °C whereas on Victrex PEEK 450G™ started from 320 °C, to ensure recording the viscosity during melt transition. The drop of viscosity during melting occurred around 300 °C for partially crosslinked PAEK-B2, around 310 °C for 0% crosslinked PAEK-B2, and around 350 °C for Victrex PEEK 450G™. The low initial viscosity of PAEK-B2 0% indicates a low MW in comparison with Victrex PEEK 450G™.

When the temperature keeps rising beyond the melting point, the melt viscosity of the 0% crosslinked PAEK-B2 first decreases then subsequently increases above 370 °C. The transition point marks the initiation of crosslinking reaction. For the partially crosslinked PAEK-B2, the melt viscosity keeps increasing after an initial shallow drop in viscosity due to remaining melt ability, resulting from the continuation of the crosslinking reaction. Eventually, partially crosslinked PAEK-B2 approaches and surpasses the similar viscosity of Victrex PEEK 450G™ in solid-state, which is a strong indication of molecular network formation, resulting in a stiff material after the test.

The rheological results prove that PAEK-B2 can maintain a low melt viscosity before the crosslinking reaction, while the final viscosity is tuneable via post heat treatment conditions. The results provide some early evidence for the feasibility of using PAEK-B2 in manufacturing CF/PAEK composites. If adopting PAEK-B2 in carbon fibre composite fabrication, a melt viscosity around 4000 Pa^.^s could be achieved at approximately 320 °C compared to 15,000 Pa^.^s when using Victrex PEEK 450G™.

**Figure 7:**
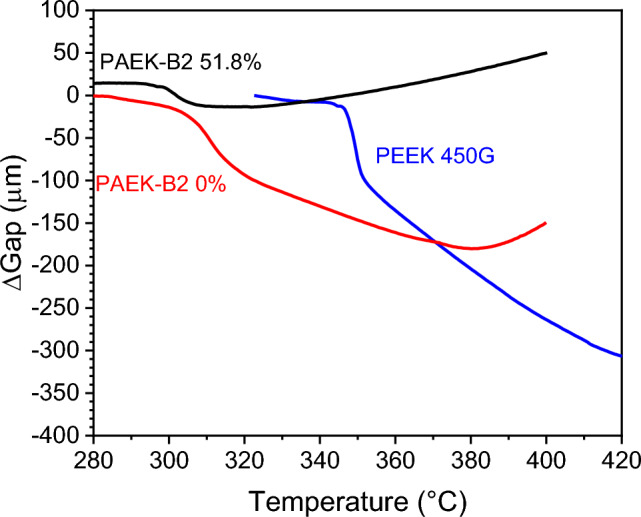
Gap change of Victrex PEEK 450G™, PAEK-B2 0% and 51.8% crosslinked, as a function of temperature during temperature sweep viscosity measurements at 5 °C/min and 1 Hz.

During the measurements between parallel plates, the constant axial force control enables tracking of the gap change of the specimens. The parallel plates’ gap of three specimens as a function of the testing temperature are displayed in Fig. 7. As expected, for non-crosslinked PAEK-B2 and Victrex PEEK 450G™, the gap is closing above melting point as the material is becoming softer, as the material is gradually squeezed out from the parallel plates. After crosslinking network forms, the disc specimen is able to maintain its shape under high temperatures, constraining the material inside the plates. Hence volume change due to thermal expansion gradually increased the gap between the plates.

### Mechanical properties

**Figure 8:**
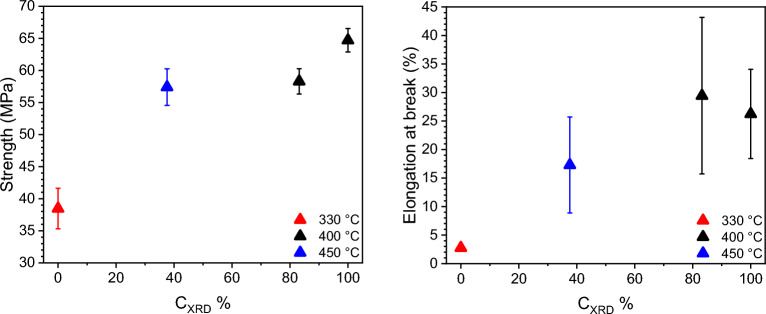
Tensile mechanical properties as a function of crosslinking degrees. Colour codes pressing temperatures.

Figure 8 presents the tensile properties as a function of the degree of crosslinking. A positive correlation can be observed. Non-crosslinked PAEK-B2 samples exhibit relatively low tensile strength and brittle failure showing a mean strength of 38.5 MPa and a mean elongation of 2.76%. Conversely, highly crosslinked PAEK-B2 pressed at 400 °C for 60 min exhibit enhanced strength and ductility, with a mean strength of 64.7 MPa and a mean elongation of 26.2%. This result suggests that increased crosslinking leads to improved mechanical properties, possibly due to molecular chain extension following the crosslinking reaction. The higher molecular weight resulting from crosslinking potentially contributes to the enhancement of both strength and ductility in PAEK-B2 samples. The 100% crosslinked PAEK-B2 has a lower strength than normally observed in conventional PEEK samples (e.g., around 100 MPa^1^) so far. This is probably partially due to different sample preparation methods, e.g. conventional PEEK data obtained from injection moulded samples whereas the PAEK-B2 samples were cut from thin compression moulded sheet. However, this could be improved to achieve comparable values by optimising the MW and the concentration of the crosslinker in the future studies.

### Recycling

In comparison with conventional thermoset composites, a distinctive feature of PAEK-b2 lies in its controllable degree of crosslinking through varying processing conditions. When not fully crosslinked, PAEK-B2 exhibits some level of crystallinity, and therefore the ability to be re-processed as a thermoplastic.

One possible approach is to mill this material into powder and diluting it with virgin PAEK. This process allows for recycling with minimal impact on the material’s properties or processing characteristics. The crosslinked material, even with a low level of retained crystallinity, may serve as a nucleation agent in virgin PAEK, which might facilitate for example the processing of slow-crystallising PAEK copolymers in injection moulding applications.

## Conclusions

This study introduced a novel class of PAEK-B2 materials that hold great potential for the development of CF/PAEK composites:

- Controlled crosslinking vs crystallisation: the degree of crosslinking in these materials is controlled by processing temperatures and holding times, showing an inverse relationship with the degree of crystallinity.
- Tailored crosslinker: the ability to add crosslinkers within the polymerisation process and tailor their response to processing temperature and time could make them suitable for other manufacturing processes.
- Impact on mechanical properties: notably, the degree of crosslinking has a significant impact on the mechanical properties. Non-crosslinked PAEK-B2 samples displayed low tensile strength and brittle failure, whereas highly crosslinked samples demonstrated increased strength and enhanced ductility.
- Rheological advantages: rheological results indicate that PAEK-B2 significantly reduce the required processing temperature and offers a broader processing window, comparing to conventional PEEK.

These findings provide valuable insights for the design and optimisation of crosslinked PAEKs, promoting their application in diverse fields such as serving as the polymeric matrix for thermoplastic composites.

## Data Availability

The data presented in this study are available on request from the corresponding author.
